# Evaluation of Low-Cost CO_2_ Sensors Using Reference Instruments and Standard Gases for Indoor Use

**DOI:** 10.3390/s24092680

**Published:** 2024-04-23

**Authors:** Qixiang Cai, Pengfei Han, Guang Pan, Chi Xu, Xiaoyu Yang, Honghui Xu, Dongde Ruan, Ning Zeng

**Affiliations:** 1 State Key Laboratory of Numerical Modeling for Atmospheric Sciences and Geophysical Fluid Dynamics, Institute of Atmospheric Physics, Chinese Academy of Sciences, Beijing 100029, China; caiqixiang@mail.iap.ac.cn; 2 Qiluzhongke Institute of Carbon Neutrality, Jinan 250100, China; 3 State Key Laboratory of Atmospheric Environment and Extreme Meteorology, Institute of Atmospheric Physics, Chinese Academy of Sciences, Beijing 100029, China; 4 Carbon Neutrality Research Center, Institute of Atmospheric Physics, Chinese Academy of Sciences, Beijing 100029, China; 5 Shandong Jinan Ecological and Environmental Monitoring Center, Jinan 250102, China; 13969150728@163.com (G.P.); jnhbjyangxiaoyu@jn.shandong.cn (X.Y.); 6 State Environmental Protection Key Laboratory of Quality Control in Environmental Monitoring, China National Environmental Monitoring Centre, Beijing 100012, China; xuchi@cnemc.cn; 7 Zhejiang Lin’an Atmospheric Background National Observation and Research Station, Hangzhou 311300, China; forsnow@126.com; 8 Zhejiang Hangzhou Ecological and Environmental Monitoring Center, Hangzhou 310012, China; sunshinefish26@163.com; 9 Department of Atmospheric and Oceanic Science, University of Maryland, College Park, MD 20742, USA; zeng@umd.edu; 10 Earth System Science Interdisciplinary Center, University of Maryland, College Park, MD 20742, USA

**Keywords:** CO_2_ monitoring, NDIR sensor, medium precision, low-cost sensor, evaluation

## Abstract

CO_2_ monitoring is important for carbon emission evaluation. Low-cost and medium-precision sensors (LCSs) have become an exploratory direction for CO_2_ observation under complex emission conditions in cities. Here, we used a calibration method that improved the accuracy of SenseAir K30 CO_2_ sensors from ±30 ppm to 0.7–4.0 ppm for a CO_2_-monitoring instrument named the SENSE-IAP, which has been used in several cities, such as in Beijing, Jinan, Fuzhou, Hangzhou, and Wuhan, in China since 2017. We conducted monthly to yearly synchronous observations using the SENSE-IAP along with reference instruments (Picarro) and standard gas to evaluate the performance of the LCSs for indoor use with relatively stable environments. The results show that the precision and accuracy of the SENSE-IAP compared to the standard gases were rather good in relatively stable indoor environments, with the short-term (daily scale) biases ranging from −0.9 to 0.2 ppm, the root mean square errors (RMSE) ranging from 0.7 to 1.6 ppm, the long-term (monthly scale) bias ranging from −1.6 to 0.5 ppm, and the RMSE ranging from 1.3 to 3.2 ppm. The accuracy of the synchronous observations with Picarro was in the same magnitude, with an RMSE of 2.0–3.0 ppm. According to our evaluation, standard instruments or reliable standard gases can be used as a reference to improve the accuracy of the SENSE-IAP. If calibrated daily using standard gases, the bias of the SENSE-IAP can be maintained within 1.0 ppm. If the standard gases are hard to access frequently, we recommend a calibration frequency of at least three months to maintain an accuracy within 3 ppm.

## 1. Introduction

As more than 70% of global fossil fuel CO_2_ emissions originate from cities [1], to achieve net-zero GHG emissions by 2050 and a 45% decrease in emissions by 2030 [2], effective CO_2_ monitoring in cities will be crucial for meeting emission reduction commitments. Several major cities in the USA and Europe have conducted carbon monitoring projects over the last decade [3,4,5,6,7,8]. Since then, some studies have evaluated the accuracy of emission inventories by relying on a CO_2_ observation network and inverse modeling approaches [7,9,10].

The emission sources of CO_2_ in cities are diverse [11,12] and have complex spatial and temporal changes [13]. Moreover, the observed concentrations are dominated by meteorological changes [14,15] and could be misinterpreted by biogenic fluxes [16]. A strategy for resolving these challenges is to establish high-density, low-cost, and medium-precision sensor (LCS) networks [17,18,19,20]. In recent decades, medium-precision carbon monitoring equipment has been used in the exploratory stage of application. Compared to high-precision instruments, such as Picarro and ABB-LGR, LCSs, which have accuracies of 1–10 ppm, have a price reduction of more than one order of magnitude (USD 5000–15,000) and can be deployed in large quantities. Such low-cost and high-density advantages make them competitive for urban carbon monitoring [21,22,23].

Currently, several cities, such as the Swiss network [22] and the California network in the USA [21,24] (Table 1), have constructed high-density CO_2_ networks using LCSs. To reveal the site-specific CO_2_ signals at most locations in Switzerland, the Carbosense CO_2_ sensor network in Switzerland includes more than 300 nodes and was constructed using Swedish SenseAir LP8 sensors (Senseair AB, Delsbo, Sweden). With calibration in a laboratory chamber, ambient colocation with a nearby reference instrument, and regular drift correction during deployment, the observation accuracy can reach between 8 and 12 ppm [22]. The Berkeley Environmental Air Quality and CO_2_ Network (BEACO_2_N) in California, USA, has approximately 40 sites, where the Vaisala CarboCap GMP343 sensors measure atmospheric CO_2_. With an in situ method for correcting biases and time-dependent drift over time, the calibrated observation accuracy is approximately 1–4 ppm [21,25]. Furthermore, the reported accuracy of BEACO_2_N reached 1.6–3.6 ppm when temperature corrections were used [24]. The medium-precision CO_2_ measuring instrument also included a LI-COR-830/850, with an accuracy between 6 and 12 ppm.

China has committed to peaking its carbon emissions before 2030 and achieving carbon neutrality before 2060 (the dual carbon goals, DCGs) [26,27,28]. To dynamically assess CO_2_ emissions from cities, the Ministry of Ecology and Environment of China issued the “Carbon monitoring and assessment pilot work program” in 2021 [29]. Since then, the carbon monitoring abilities of cities and provinces in China have greatly increased.

Since 2017, the Institute of Atmospheric Physics, Chinese Academy of Sciences, has established a network with more than 120 sites using LCSs for CO_2_ monitoring, mostly in the city of Beijing and surrounding regions [30]. We made efforts in the environmental calibration of the instrument to improve the accuracy of SenseAir K30 CO_2_ sensors from ±30 ppm to 0.8–4 ppm [31,32]. However, compared to the stability of high-precision instruments, LCSs are susceptible to time-dependent drift over time as well as to environmental variables [24,33,34,35]. Thus, LCSs are usually taken to a laboratory for regular calibration or for in situ field calibration for biases.

In this study, we conducted synchronous observations of our environment-calibrated LCSs for a few weeks to more than one year at four different sites under typical use conditions. The LCSs were compared to high-precision Picarro instruments, and standard gases were traceable to the WMO X2007 scale. Our comparison provides a basic understanding and evaluation of the performance of our LCSs. The use of high-precision instruments and standard gas tanks also helps us gain a basis for short-term and long-term time-dependent drift calibration methods that can improve the accuracy of urban CO_2_ networks using LCSs.

## 2. Materials and Methods

### 2.1. Sensor Deployment

**Figure 1:**
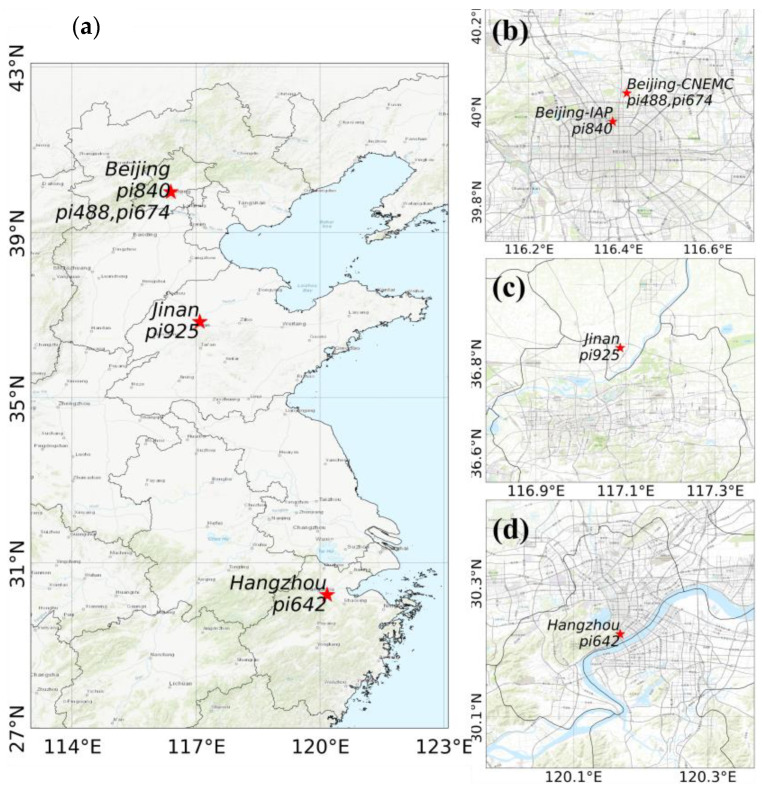
(**a**) Map of the four deployment sites; (**b**) Beijing sites: Institute of Atmospheric Physics (Beijing-IAP) and China National Environment Monitoring Center (Beijing-CNEMC); (**c**) Jinan site, Shandong Province; and (**d**) Hangzhou site, Zhejiang Province.

The LCSs were deployed in the following three cities (Figure 1a): Beijing (Figure 1b), Jinan in Shandong Province (Figure 1c), and Hangzhou in Zhejiang Province (Figure 1d). They were compared with a high-precision instrument or standard gas. The Beijing sites were located at the Institute of Atmospheric Physics, Chinese Academy of Sciences (Beijing-IAP, 39.39 N, 116.39 E, 62 m) and the China National Environment Monitoring Center (Beijing-CNEMC, 40.04 N, 116.42 E), in the central area of the city, which has a high population density and is greatly affected by traffic emissions. The Jinan site was in the suburbs of an urban area (36.83 N, 117.09 E, 44 m) featuring a low population density and low emissions from transportation and industrial parks. The Hangzhou Mount Mantou station (30.23 N, 120.16 E, 43.2 m a.s.l.) is located at the convergence of the West Lake scenic area and residential areas in the south of city, where there is a high population density and influence of human activities.

The precision and specificity of the reading accuracy of the sensors and instruments at the four sites are listed in Table 2. For our LCSs, we used a non-dispersive infrared (NDIR) CO_2_ measurement (SenseAir K30, Senseair AB, Delsbo, Sweden), which has a ±30 ppm raw accuracy [36]. Three K30s were put in one instrument with a Bosch BME680 sensor to record the temperature, humidity, and air pressure [31,32]. This integrated instrument with environmental calibration is called the SENSE-IAP. We used a cavity ring-down spectrometer (Picarro G2301/G4301, Picarro, Santa Clara, CA, USA) as the high-precision instrument for the CO_2_ measurements [37,38]. The precision and accuracy of the Picarro instrument were better than 0.1 ppm [39]. The standard gas tanks used at all four sites were obtained from the Meteorological Observation Center of the China Meteorological Administration (MOC/CMA) and traceable to the World Meteorological Organization (WMO) X2007 scale.

To ensure the long-term synchronous observation of the LCSs and high-precision instruments, in their deployment, we made an effort to ensure that the two sets of instruments measured the same gas mass. That is, we ensured that the differences in the instrument observation values only came from the effects of temperature, humidity, air pressure, and the concentration span, which can be adjusted by calibration methods. What is more, to evaluate the gradual change in the systematic bias of low-cost sensors in long-term deployment, the synchronous observation instruments were all in a relatively stable indoor environment. Although the outdoor air CO_2_ concentration extracted by the pump exhibited significant diurnal variations (Figure S1a,c), the temperature and humidity of the pre-processed air were basically stable (Figure S1b,d,f).

**Figure 2:**
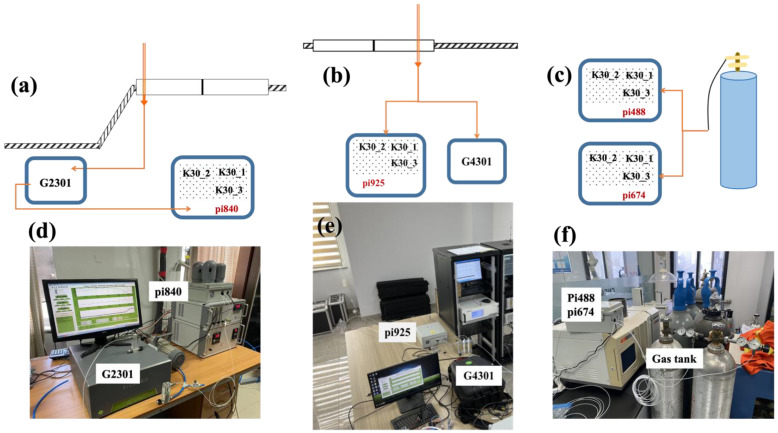
Synchronous observation system of the LCSs and the Picarro instrument. Diagram (**a**–**c**) of gas flow for the LCSs and Picarro instrument. Photographs (**d**–**f**) of the instrument installation. (**a**,**d**) Beijing-IAP, (**b**,**e**) Jinan, and (**c**,**f**) Beijing-CNEMC.

At the Beijing-IAP, Jinan, and Hangzhou sites, the ambient air was drawn by a pump from outside the window, and the intake was linked through a pipe with a particulate matter filter and a water dryer. As shown in Figure 2a,d, to assess the precision and accuracy of the SENSE-IAP, at the site of Beijing-IAP, excess gas was discharged from the outlet of the Picarro system and connected to sensors on the SENSE-IAP (number pi840). The ambient air at the Jinan site was diverted through a three-way valve to the SENSE-IAP (number pi925) and Picarro G4301 (Figure 2b,e). The long indoor pipe caused the temperature of the air gas to approach the indoor temperature controlled by the air conditioner. At the Beijing-IAP and Jinan sites, the Picarro analyzer was calibrated every month by high-pressure standard gases.

For the two SENSE-IAPs (numbers pi488 and pi674) deployed at Beijing-CNEMC, the standard gas from the gas tank directly flowed into the sensor of the SENSE-IAP through pipes (Figure 2c,f). We also deployed the SENSE-IAP (number pi642) at Hangzhou using a deployment method similar to that used for Beijing-IAP. The difference is that we only analyzed the CO_2_ concentration observed by the SENSE-IAP when the standard gas was introduced every six hours.

### 2.2. Sensor Calibration and Evaluation Parameters

We developed a calibration system to substantially improve the CO_2_ accuracy of the K30 sensor. To reduce the background or white noise, we excluded anomalous observation values based on the 3-σ principle every minute. After calibrating for the sensitivity of temperature, humidity, and pressure effects in the laboratory [35], the accuracy increased to 1–4 ppm in comparison to that of Picarro [31]. We adjusted the calibration of the span and system bias before observation and adjusted the gradual time-dependent drift (in ppm/day) using the following formula:

(1) $$C_{Cor}^{drift}=C-∆C_{drift}$$

where Δ*C_drift_* is the bias between the concentration *C* measured by the instrument and the standard concentration *C*_0_ at the end of the time-dependent drift. The end time of drift is the time when the slope of the drift tended toward stability or any time when the sensors needed to be corrected.$C_{Cor}^{drift}$ is the long-term drift-calibrated CO_2_ concentration.

The following two parameters were used to evaluate the precision and accuracy of the SENSE-IAP. The root mean square error (RMSE) (2) was used to evaluate the precision, and the bias (3) was used to determine the measurement accuracy of the SENSE-IAP compared with that of the Picarro standard gas concentration.

(2) $$\mathrm{RMSE}=\sqrt{\frac{\sum {\left(C-C_{0}\right)}^{2}}{n-1}}$$

(3) $$\mathrm{Bias}=C-C_{0}$$

where *C* is the CO_2_ concentration measured by the SENSE-IAP, and *C*_0_ is the standard concentration measured by Picarro or the concentration of standard gas.

## 3. Results

### 3.1. Daily to Monthly Comparisons with Standard Gases

**Figure 3:**
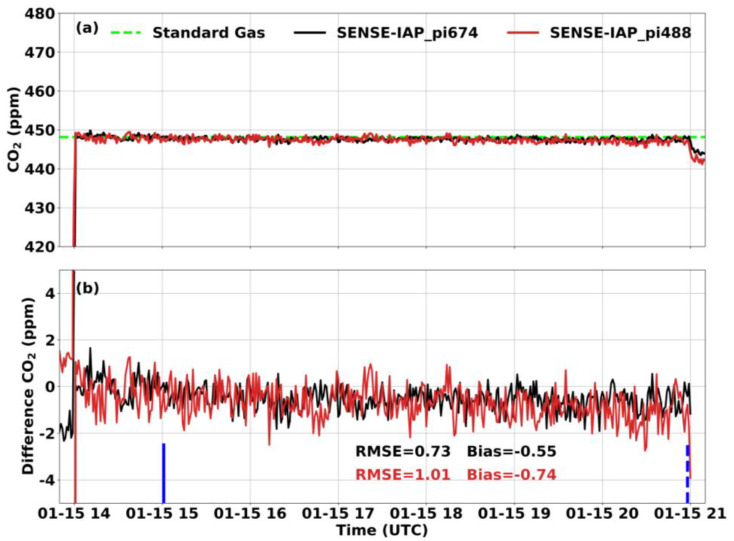
Comparison between the CO_2_ concentrations per minute measured by the SENSE-IAP and the CO_2_ concentrations of standard gas at Beijing-CNEMC on 15 January 2022. The blue lines show the time span of the sampling data. (**a**) The mean of the three sensors on one SENSE-IAP instrument; the plot for each sensor is shown in Figures S2 and S3. (**b**) The time series of the difference with standard gas. Black is the mean of the three sensors on pi674, and gray is the mean of the sensors on pi488.

At the Beijing-CNEMC site, we conducted observations of the SENSE-IAPs for both short-term tests (six hours) and long-term tests (seven weeks) in the months of January and February 2022. Throughout the experiment, the temperature in the laboratory was controlled at 23–25 °C by air conditioning. And the gas from the standard gas tank was similar to dry gas with very low water content. For short-term observations, the SENSE-IAP monitored the CO_2_ concentration from the gas tank for seven continuous hours, and the data from the first hour were not calculated due to the need for stable ventilation. During the continuous short-term observation of the standard gas, the SENSE-IAPs exhibited a bias of −0.56 ± 0.38 ppm compared to the standard gas, with an RMSE of 1.16 ± 0.28 ppm (Figure 3 and Table 3).

**Figure 4:**
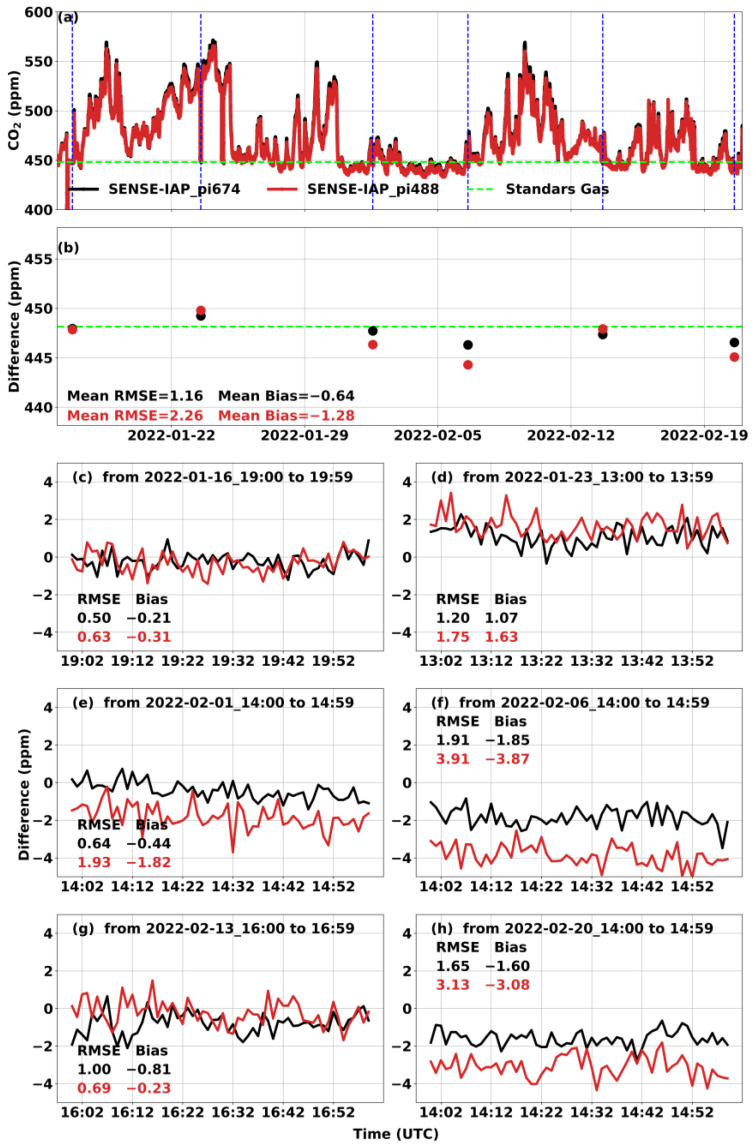
Comparison between the CO_2_ concentrations measured by the SENSE-IAP and the CO_2_ concentrations of standard gas at Beijing-CNEMC in January and February 2022. (**a**) The time series of CO_2_ per minute in the whole measurement period; the blue dashed lines mark one hour of the standard gas measurement per week, and the green dashed lines mark the concentration of standard gas. (**b**) The points are the hourly means of values during one hour of standard gas measurement per week. (**c**–**h**) The time series of the difference in the standard gas in each week. Black is the mean of the three sensors on pi674, and red is the mean of the sensors on pi488. The plots for all the sensors in each SENSE-IAP instrument are shown in Figures S4 and S5.

For the long-term observation, the CO_2_ concentration in the gas tank of the SENSE-IAP was monitored for one hour per week. Outside of the specific one-hour period of the standard gas measurement per week, the SENSE-IAP measured the ambient CO_2_ concentration. The hourly mean CO_2_ concentrations monitored over six weeks compared to those of the standard gas are shown as points in Figure 4b. For all six sensors, the bias was −0.56 ± 0.38 ppm, and the RMSE was 2.08 ± 0.68 ppm (Figure 4a,b and Table 3). For each standard gas measurement, the bias was in the range of −3.87–1.63 ppm, while the RMSE was in the range of 0.5–3.91 ppm (Figure 4c–h). The long-term observations also show a time-dependent drift during the testing period of approximately 1.5 months. As shown in Table 3, the long-term drift trends for all six sensors were −0.09 ± 0.03 ppm per day or −3.73 ± 1.16 ppb per hour.

### 3.2. Daily to Yearly Comparisons with Standard Gases

Synchronous monitoring at the Hangzhou site revealed longer comparative observations for the SENSE-IAP and standard gas. The temperature and relative humidity (RH) ranges of the monitoring period were mainly between 25 and 35 °C and 20 and 40% RH, respectively. We analyzed the accuracy and stability of the SENSE-IAP based on the difference between the concentration measured by the SENSE-IAP and the standard gas used during the Picarro calibration period every 6 h.

**Figure 5:**
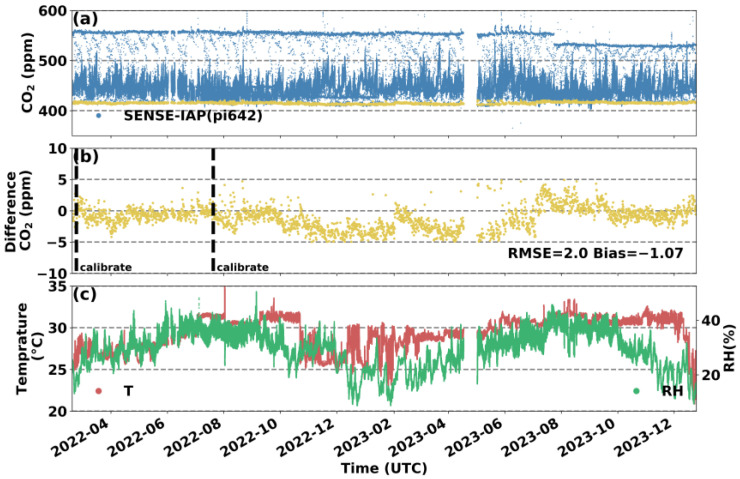
Comparison between the minute CO_2_ concentrations measured by the SENSE-IAP and the CO_2_ concentrations of standard gas at the Hangzhou site from 18 February 2022 to 25 December 2023. (**a**) Time series of the CO_2_ concentrations; the blue points are the means of the CO_2_ concentrations measured by the three sensors on the SENSE-IAP; the yellow points indicate the time points when the standard gas was introduced; and (**b**) the difference between the measured CO_2_ and standard gas during the period when the gas was introduced; the points are consistent with (**a**) in terms of time. (**c**) The temperature and RH of the SENSE-IAP.

Figure 5 shows the mean CO_2_ concentration measured by the three K30s of the SENSE-IAP at Hangzhou. The yellow points are the values per minute obtained during the Picarro calibration every 6 h. The RMSE of the SENSE-IAP relative to the introduced standard gas was 2.0 ppm, with a mean bias of −1.1 ppm for the entire 22 months of monitoring, with only one calibration after approximately 5 months of deployment for K30 and K30_3. For K30_2, long-term drift occurred 6 months after the first calibration (at 2023-02), with a daily drift of 0.1 ppm (Figure S6).

### 3.3. Monthly Comparisons to Standard Instruments (Picarro)

**Figure 6:**
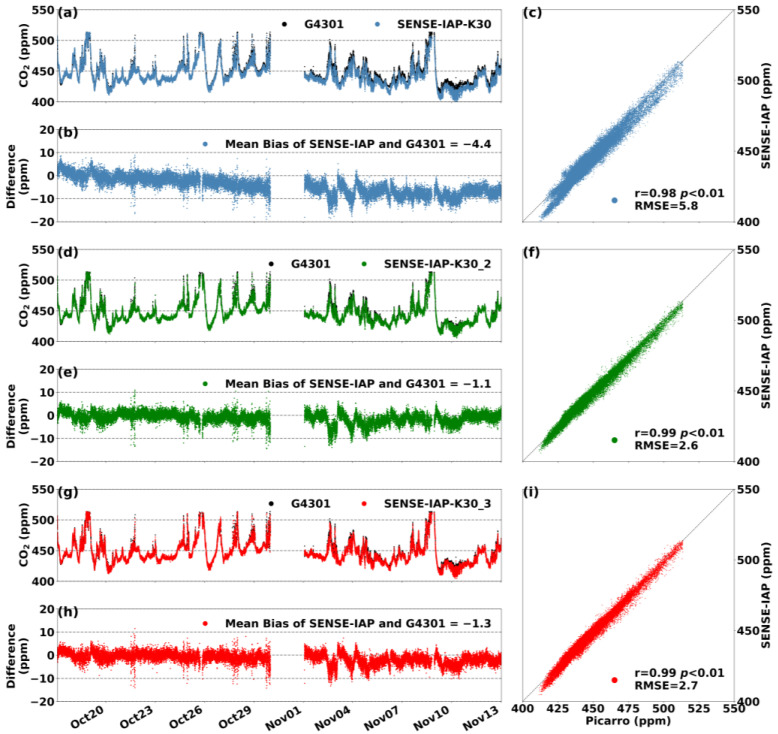
(**a**,**d**,**g**) Comparisons of the CO_2_ concentrations per minute measured by three sensors (the SENSE-IAP and the Picarro system) at the Jinan site from 17 October to 17 November in 2023. (**b**,**e**,**h**) The time series of ΔCO_2_, and (**c**,**f**,**i**) the scatterplots of the SENSE-IAP and Picarro. (**a**–**c**) The first sensor, K30, (**d**–**f**) the second sensor, K30_2, and (**g**–**i**) the third sensor, K30_3.

At the Jinan site, the SENSE-IAP was co-localized (side-by-side) with G4301 for 4 weeks in an environment in which the diurnal temperature and humidity changes were not apparent (Figure S7c). Throughout the instrument monitoring period, the temperature and humidity ranged from 25 to 35 °C and from 0 to 40% RH, respectively. After environmental corrections, the RMSE of the SENSE-IAP was generally consistent with that of the Picarro system, ranging from 2.6 to 5.8 ppm. Figure 6b shows the difference in the CO_2_ concentration between the SENSE-IAP and Picarro systems (ΔCO_2_) during the observation period. The K30 sensor error (Figure 6b) mainly originated from a mean bias of −4.4 ppm, with a slope of 0.37 ppm/day (<0.02 ppm/h). For the other two sensors (K30_2 and K39_3), the main source of error occurred at the time at which the concentration significantly changed. If a linear correction was performed (Section 2.2) on sensor K30 during the start/end point of the observation period, the RMSE could be improved to at least 3.0 ppm (Figure S7).

### 3.4. Yearly Comparison to Standard Instruments

**Figure 7:**
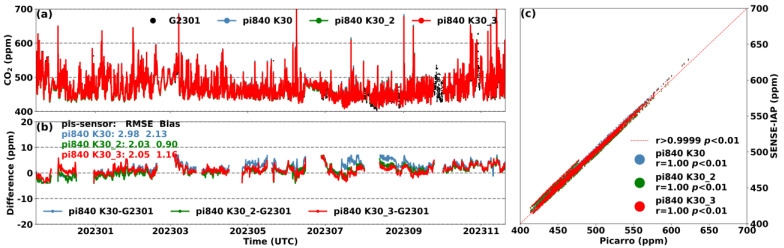
(**a**) Comparison between the hourly CO_2_ concentrations measured by the three sensors on the SENSE-IAP and the Picarro system at the Beijing-IAP site from November 2022 to November 2023; (**b**) time series of ΔCO_2_; (**c**) scatter plot of the SENSE-IAP and Picarro.

Figure 7 shows the results of the environment-corrected and long-term drift-adjusted SENSE-IAP at the Beijing-IAP site compared to those of the Picarro system. Due to the fact that the sensor of the SENSE-IAP measured the excess gas discharged from the outlet of the Picarro system, the diurnal variations in the temperature and humidity of the air were no longer significant since they were controlled by the Picarro system (Figure 1b). Throughout the observation period, the temperature and humidity ranged from 20 to 35 °C and from 10 to 20% RH, respectively (Figure S8c). All three sensors exhibited long-term downward drifts of up to −16 ppm after half a year and up to −24.4 ppm after one year of deployment. For K30s on pi840, the ΔCO_2_ showed a continuous downward trend over the past 12 months, with RMSEs of 24.5, 9.9, and 14.6 ppm, respectively (Figure S8), and the drift trend became significantly unstable over time. We adjusted the long-term drift trends of the three sensors during the observation period from November 2022 to November 2023 based on Function (1) in Section 2.2. The corrected CO_2_ concentrations from the three sensors were consistent with those of the Picarro system, with a bias ranging from 0.9 to 2.1 ppm (Figure 7). Our correction method improved the RMSE from 14.3–15.4 ppm of the sensor manufacturer’s raw data (SenseAir, Figure S9) to 2.0–3.0 ppm (SENSE-IAP, Figure 7).

Among the 15 sensors deployed in this study, 80% of the sensors exhibited a drift trend of less than 0.1 ppm per day, with the largest drift trend occurring at 0.4 ppm per day (Table 4). From the perspective of the drift degree, a significant bias of 5 ppm (approximately 1% of the ambient CO_2_ concentration) generally occurred within 1.5–3 months. Only one sensor at the Jinan site showed significant bias after half a month. The Hangzhou and Jinan sites each had one sensor that may have experienced significant drift after more than one year. Such long-term drift for the SENSE-IAP can be detected by the Picarro or standard gas when there is no reference instrument, and thus, can be corrected through postprocessing programs.

## 4. Conclusions

The environmental calibration methods that determined the specific parameters for the temperature and humidity for each sensor and the relatively stable indoor environment removed the effects of temperature and humidity fluctuations on the NDIR absorption sensors, and improved the accuracy of the SenseAir K30 sensors from ±30 ppm to 0.7–4.0 ppm for the SENSE-IAP.

We conducted monthly to yearly evaluations of low-cost CO_2_ sensors using both Picarro as a reference instrument and standard gas. The precision and accuracy of the SENSE-IAP for the short-term and long-term comparisons with standard gas had biases of −1.28 to −0.55 ppm and RMSEs ranging from 0.73 to 2.5 ppm. The relatively high precision and accuracy were due to the relatively stable indoor environment where the instruments were deployed (Figure S1b,d,f), which were typical working conditions for regular environmental monitoring work in an air-conditioned building. Nevertheless, the SENSE-IAP exhibited an ominous long-term drift of −0.09 ± 0.03 ppm per day or −3.73 ± 1.16 ppb per hour.

For the synchronous observation with Picarro, the performance of the SENSE-IAP was in the same magnitude, with a bias of −1.2–2.1 ppm and an RMSE of 2.0–3.0 ppm. The overall observation error of the SENSE-IAP after long-term drift calibration was less than 1% of the ambient CO_2_ concentration after more than one year of deployment.

Regarding long-term deviations, the time-dependent drift over time of all 15 sensors at the four sites exhibited a drift trend ranging from less than −0.01 to 0.4 ppm per day. Thus, a significant bias of 5 ppm (approximately 1% of the ambient CO_2_ concentration) typically occurred in 1.5–3 months; therefore, the long-term drift calibration frequency should be no longer than 3 months. For observations over a year, standard instruments and reliable concentration values from standard gas (which, in most cases, are much easier to access) are the two main references that are available for calibrations.

## Figures and Tables

**Table 1:** Comparisons of the precision and price of medium-precision CO_2_ measuring instruments.

	Region	Raw Accuracy (ppm)	Corrected Hourly Accuracy (ppm)	Cost Estimated (Thousand USD)	Sensor/Instrument
Carbosense CO_2_ sensor network	Switzerland	±50	6.8–13.9	4–7	LP8
BEACO_2_N	California, USA	±3 ppm + 1% reading	1.6–3.6	14	GMP343
LI-COR	USA	6–12	6–12	11–14	LI-830/850
SENSE-IAP	Beijing, Jinan, etc., China	±30	0.7–3.3	4–7	K30

**Table 2:** Sensor and instrument parameters for the four sites.

Station	Intake Height	SENSE-IAP	High-Precision Picarro					
			Analyzer	Precision in 5 min (ppm)	Maximum Drift over 24 h		Calibration Frequency	Evaluation Period of Synchronous Observation
Beijing-IAP	6 stories high (18 m)	3 K30 sensors	G2301	0.025	0.12 ppm		1 month	12 months
Jinan	5 stories high (15 m)	3 K30 sensors	G4301	0.04 ppm + 0.02% value	0.5 ppm		1 month	4 weeks
Station	Intake Height	SENSE-IAP	Frequency of verification using standard gas			Evaluation period of synchronous observation		
Beijing-CNEMC	Not applicable	6 K30 sensors	1 week			6 weeks		
Hangzhou	15m	3 K30 sensors	6 h			22 months		

**Table 3:** Performance of the six sensors on two SENSE-IAP instruments (unit: ppm).

Instrument		pi674			pi488		
Sensors		s1	s2	s3	s1	s2	s3
Short-term (Daily scale) parameters	Bias	−0.5	0.2	−0.8	−0.8	−0.6	−0.9
	RMSE	1.0	0.7	1.3	1.1	1.3	1.6
Long-term (Monthly scale) parameters	Bias	0.5	−0.9	−1.4	−1.6	−1.2	−1.1
	RMSE	1.6	1.3	2.7	3.2	2.1	1.6
	Daily drift (ppm)	−0.06	−0.06	−0.14	−0.1	−0.09	−0.09
	Hourly drift (ppb)	−2.4	−2.3	−5.8	−4.1	−3.9	−3.8

**Table 4:** The time-dependent drift of sensors at 4 sites (unit: ppm).

Site	Hangzhou			Jinan			BJ-1 ^1^			BJ-2 ^1^			BJ-3 ^2^		
Sensor	s1	s2	s3	s1	s2	s3	s1	s2	s3	s1	s2	s3	s1	s2	s3
Monthly drift (ppm/month)	−0.3		−3	−12	−0.3		−3.4	−1.5		−1.7		−4	−2	−0.8	−1.2
Daily drift (ppm/day)	<−0.01		−0.1	−0.4	<−0.01		−0.08	−0.05		−0.06		−0.1	−0.07	−0.03	−0.04

^1^ BJ-1 and BJ-2 are the SENSE-IAPs at Beijing-CNEMC. ^2^ BJ-3 is the SENSE-IAP at Beijing-IAP.

## Data Availability

The data used to generate the figures in this manuscript are available upon reasonable request to the corresponding authors.

## Supplementary Materials

The following supporting information can be downloaded at: https://www.mdpi.com/article/10.3390/s24092680/s1, Figure S1: (a–f) Diurnal variations in CO2 concentration, temperature, and humidity measured by the SENSE-IAP at three sites. (g–h) The wind rose plots display the wind speed and direction during the observation period at Beijing-IAP and Jinan. Figure S2: Comparison between the CO2 concentrations per minute measured by the SENSE-IAP and the CO2 concentrations of standard gas at Beijing-CNEMC on 15 January 2022. The blue lines show the time span of the sampling data. (a) The data from the three sensors on pi674; (b) the time series of the differences in standard gas; Figure S3: Comparison between the CO2 concentrations per minute measured by the SENSE-IAP and the CO2 concentrations of standard gas at Beijing-CNEMC on 15 January 2022. The blue lines show the time span of the sampling data. (a) The data from the three sensors on pi488; (b) the time series of the differences in standard gas; Figure S4: Comparison between the CO2 concentrations measured by the SENSE-IAP and the CO2 concentrations of standard gas at Beijing-CNEMC in January and February 2022. (a) The time series of CO2 per minute in the whole measurement period; the blue dashed lines mark the one-hour of the standard gas measurement per week, and the green dashed lines mark the concentration of standard gas. (b) The points are the hourly mean of values during the one hour of standard gas measurement per week. (c–h) The time series of the difference in standard gas each week. The blue, green, and red colors indicate the three sensors on pi674; Figure S5: Comparison between the CO2 concentrations measured by the SENSE-IAP and the CO2 concentrations of standard gas at Beijing-CNEMC in January and February 2022. (a) The time series of CO_2_ per minute in the whole measurement period; the blue dashed lines mark the one hour of the standard gas measurement per week, and the green dashed lines mark the concentration of standard gas. (b) The points are the hourly mean of values during the one hour of standard gas measurement per week. (c–h) The time series of the difference in standard gas each week. The blue, green, and red colors indicate the three sensors on pi488.; Figure S6: Comparison between the minute CO_2_ concentrations measured by the SENSE-IAP and the CO_2_ concentrations of standard gas at the Hangzhou site from 18 February, 2022 to 25 December, 2023, (a) Time series of the CO_2_ concentrations; the blue points are for the first sensor, K30, the green points are for the second sensor, K30_2, and the red points are the third sensor, K30_3. (b) The difference in the measured CO_2_ and standard gas during the period when the gas was introduced; the points are consistent with (a) in terms of time. (c) The temperature and TH of the SENSE-IAP.; Figure S7: (a) Comparison between the minute CO_2_ concentrations measured by the mean of the three sensors of the SENSE-IAP and the Picarro system at the Jinan site from 17 October to 17 November, 2023; (b) the time series of *ΔCO_2_*; (c) the temperature and RH of the SENSE-IAP; (d) the scatter plot of the SENSE-IAP and Picarro.; Figure S8: (a) Comparison between the hourly CO_2_ concentrations measured by the three sensors on the SENSE-IAP and the Picarro system at Beijing-IAP from November 2022 to November 2023; (b) the time series of ΔCO_2_ before long-term drift calibration; (c) the temperature and RH of the SENSE-IAP, (d) scatter plot of the SENSE-IAP and Picarro.; Figure S9: (a) Comparison between the hourly CO_2_ concentrations measured by the three sensors on the SenseAir and the Picarro systems at Beijing-IAP from November 2022 to November 2023; (b) the time series of ΔCO_2_ before long-term drift calibration; (c) scatter plot of SenseAir and Picarro.
