# Supplementary material for: Evaluation of Low-Cost CO2 Sensors Using Reference Instruments and Standard Gases for Indoor Use
Source: Sensors (Basel). 2024 Apr 23;24(9):2680. doi: 10.3390/s24092680 (PMC11085240; doi:10.3390/s24092680)
Supplement: Supplementary file 1 [file sensors-24-02680-s001.zip › sensors-2938243-supplementary.pdf]

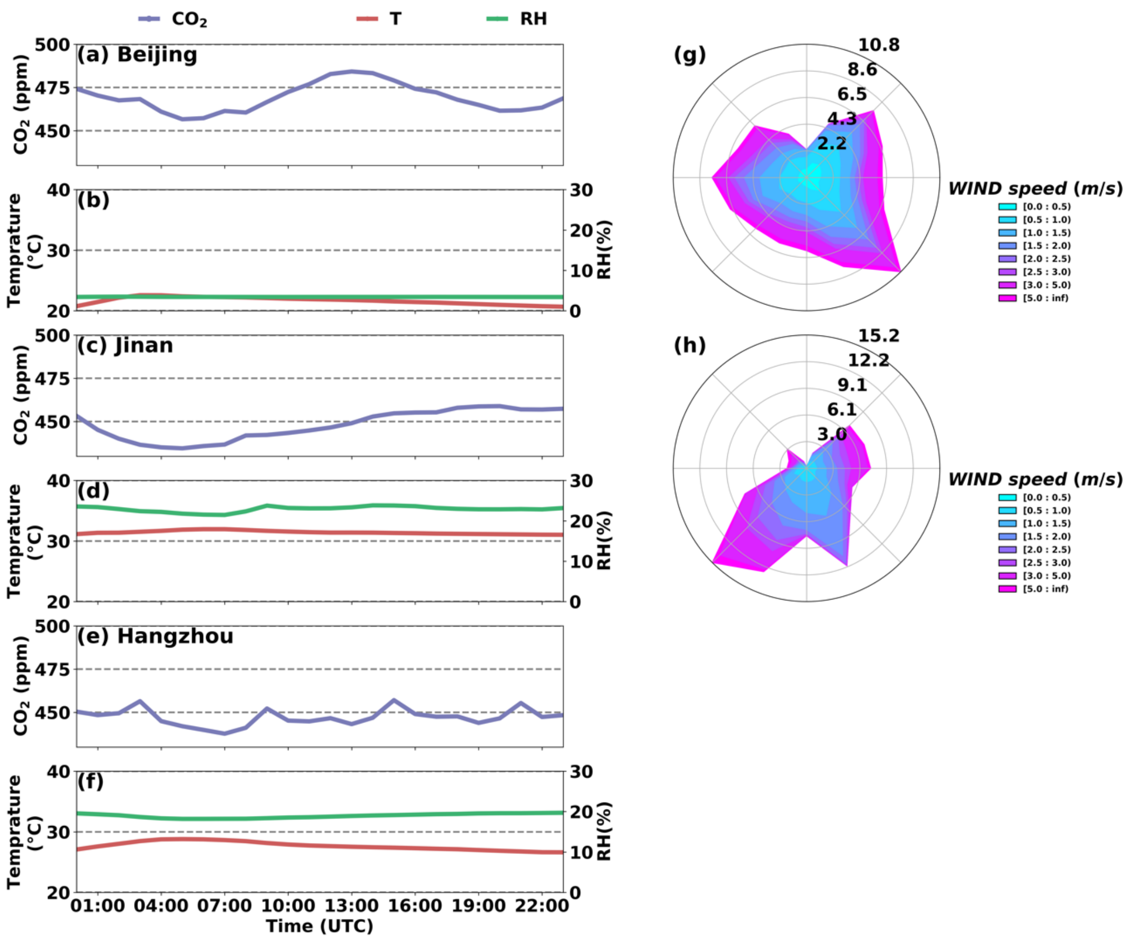

Figure S1: (a-f) Diurnal variations in the CO2 concentration, temperature, and humidity measured by the SENSE-IAP at three sites. (g-h) The wind rose plots display the wind speed and direction during the observation period at the Beijing-IAP and Jinan sites.

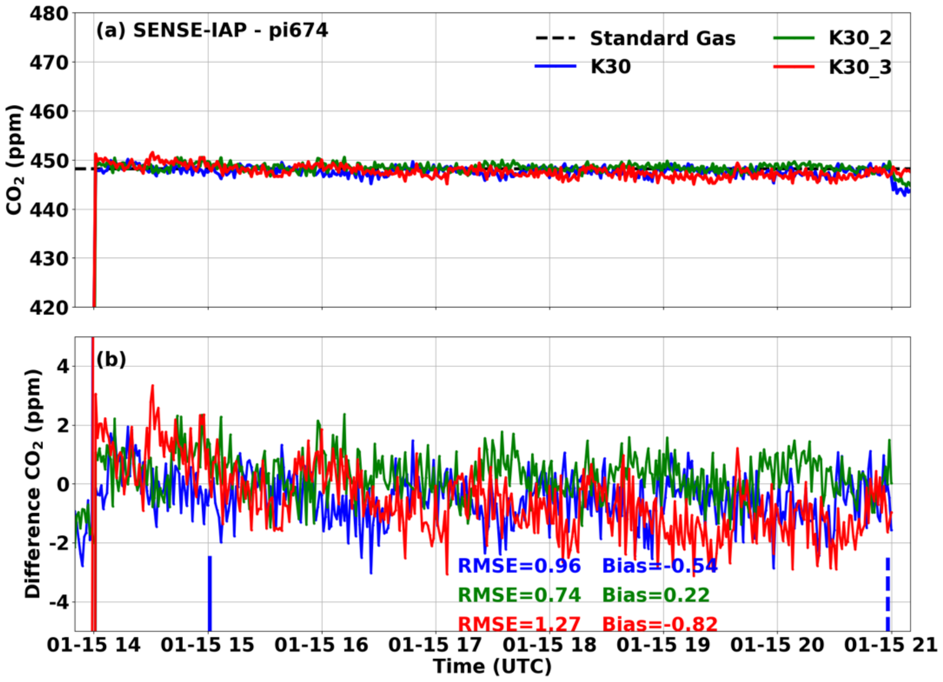

Figure S2: Comparison between the CO_2 concentrations per minute measured by the SENSE-IAP and the CO_2 concentrations of standard gas at Beijing-CNEMC on 15 January 2022. The blue lines show the time span of the sampling data. (a) The data from the three sensors on pi674; (b) the time series of the difference in standard gas.

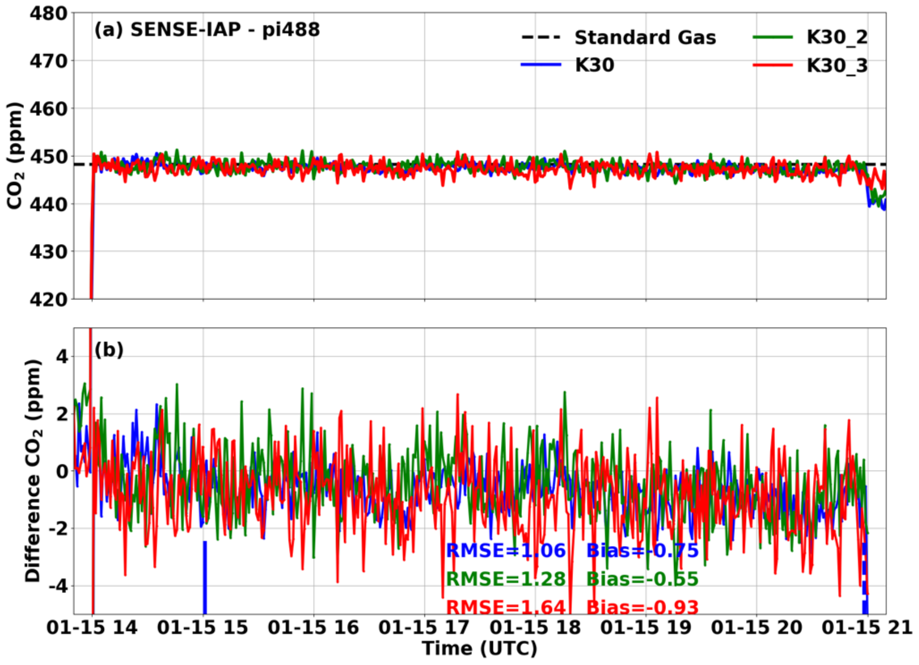

Figure S3: Comparison between the CO_2 concentrations per minute measured by the SENSE-IAP and the CO_2 concentrations of standard gas at Beijing-CNEMC on 15 January 2022. The blue lines show the time span of the sampling data. (a) The data from the three sensors on pi488; (b) the time series of the difference in standard gas.

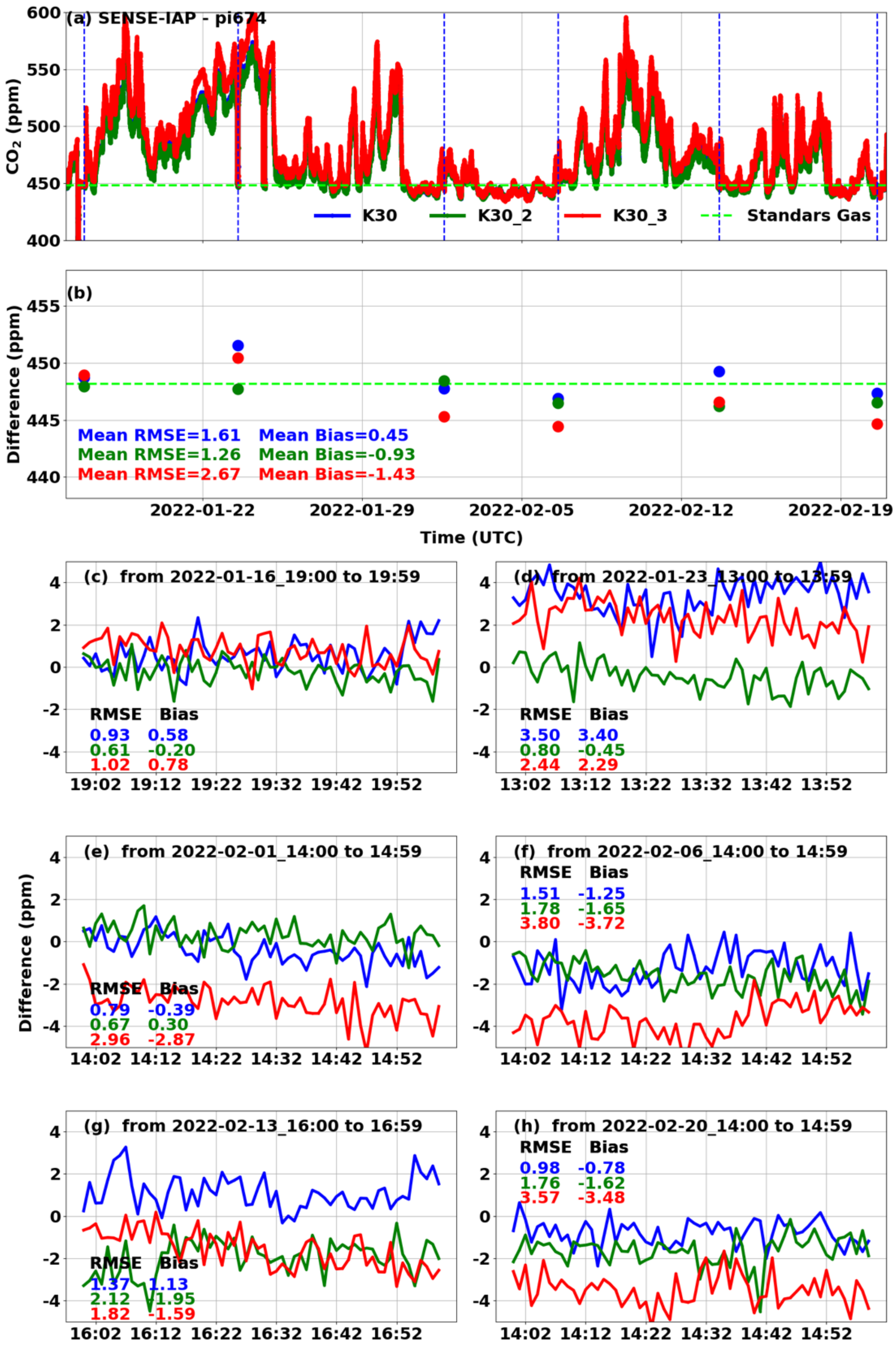

Figure S4: Comparison between the CO_2 concentrations measured by the SENSE-IAP and the CO_2 concentrations of standard gas at Beijing-CNEMC in January and February 2022. (a) The time series of CO_2 per minute in the whole measurement period; the blue dashed lines mark the one hour of the standard gas measurement per week, and the green dashed lines mark the concentration of standard gas. (b) The points are the hourly means of values during the one hour of standard gas measurement per week. (c-h) The time series of the difference in standard gas in each week. The blue, green, and red colors indicate the three sensors on pi674.

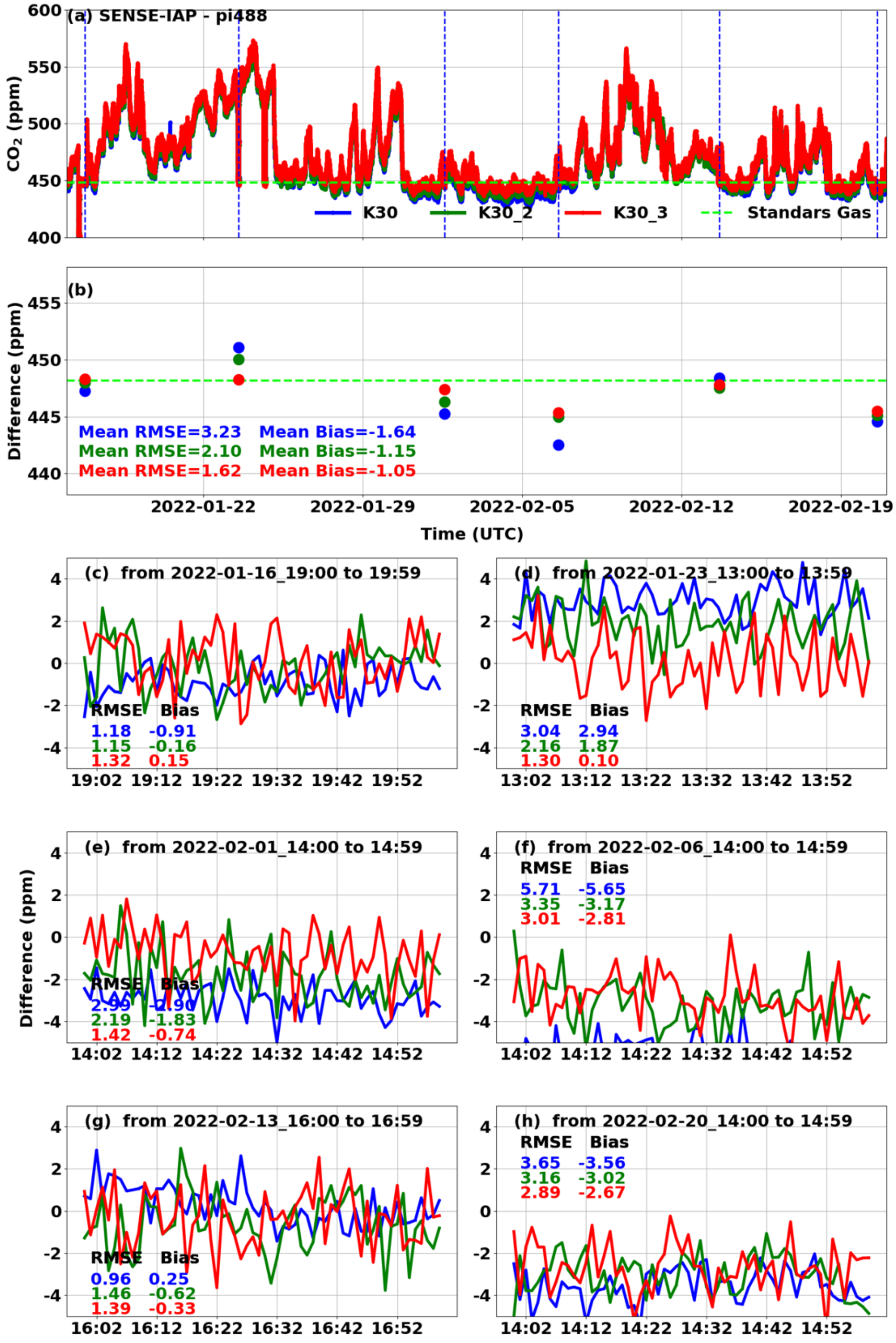

Figure S5: Comparison between the CO_2 concentrations measured by the SENSE-IAP and the CO_2 concentrations of standard gas at Beijing-CNEMC in January and February 2022. (a) The time series of CO_2 per minute in the whole measurement period; the blue dashed lines mark the one hour of the standard gas measurement per week, and the green dashed lines mark the concentration of standard gas. (b) The points are the hourly means of values during the one hour of standard gas measurement

per week. (c-h) The time series of the difference in standard gas in each week. The blue, green, and red colors indicate the three sensors on pi488.

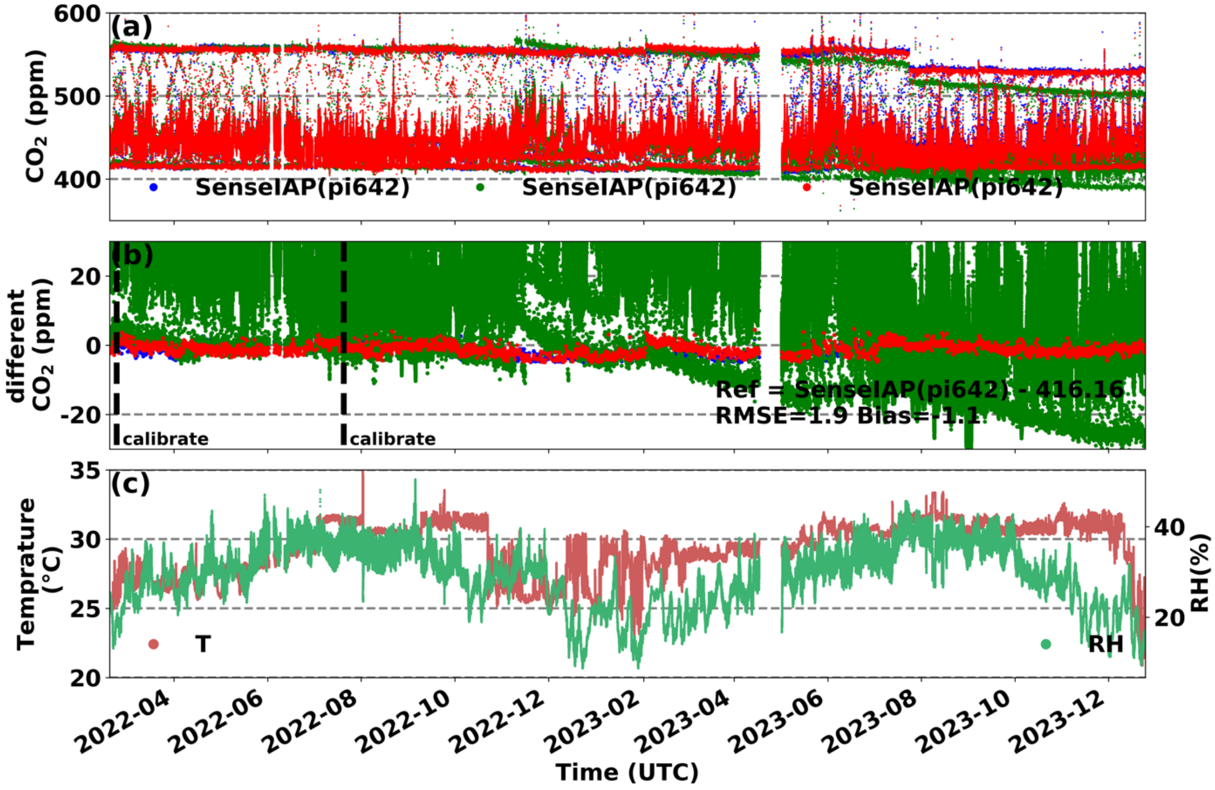

Figure S6: Comparison between the minute CO_2 concentrations measured by the SENSE-IAP and the CO_2 concentrations of standard gas at Hangzhou site from 18 February, 2022 to 25 December, 2023, (a) Time series of the CO_2 concentrations; the blue points are for the first sensor, K30, the green points are for the second sensor, K30_2, and the red points are the third sensor, K30_3. (b) The difference in the measured CO_2 and standard gas during the period when the gas was introduced; the points are consistent with (a) in terms of time. (c) The temperature and TH of the SENSE-IAP.

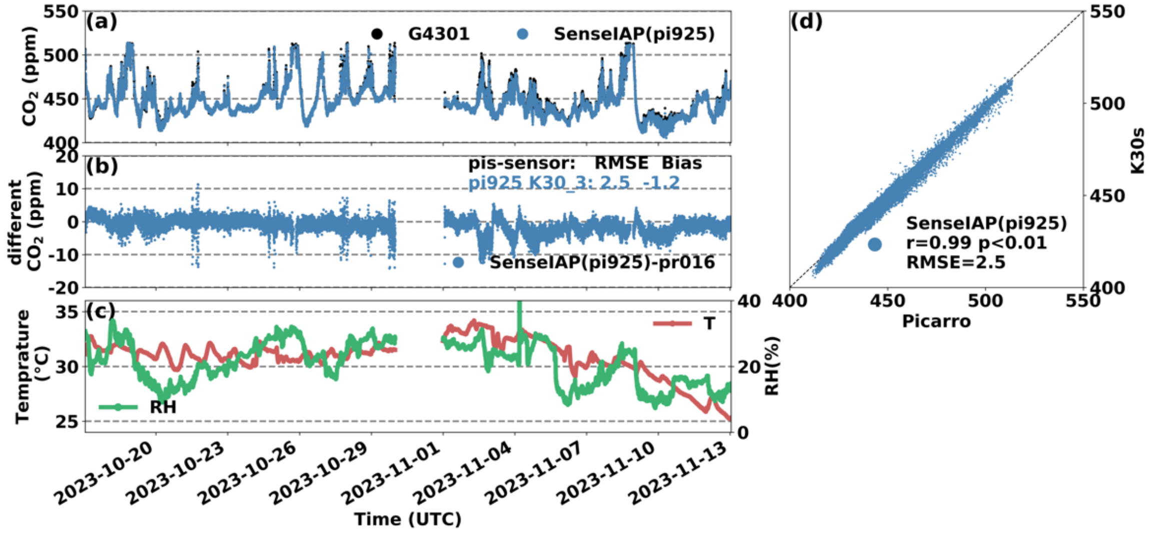

Figure S7: (a) Comparison between the minute CO_2 concentrations measured by the mean of the three sensors of the SENSE-IAP and Picarro system at the Jinan site from 17 October to 17 November, 2023; (b) the time series of ΔCO_2, (c) temperature and RH of the SENSE-IAP; (d) the scatter plot of the SENSE-IAP and Picarro.

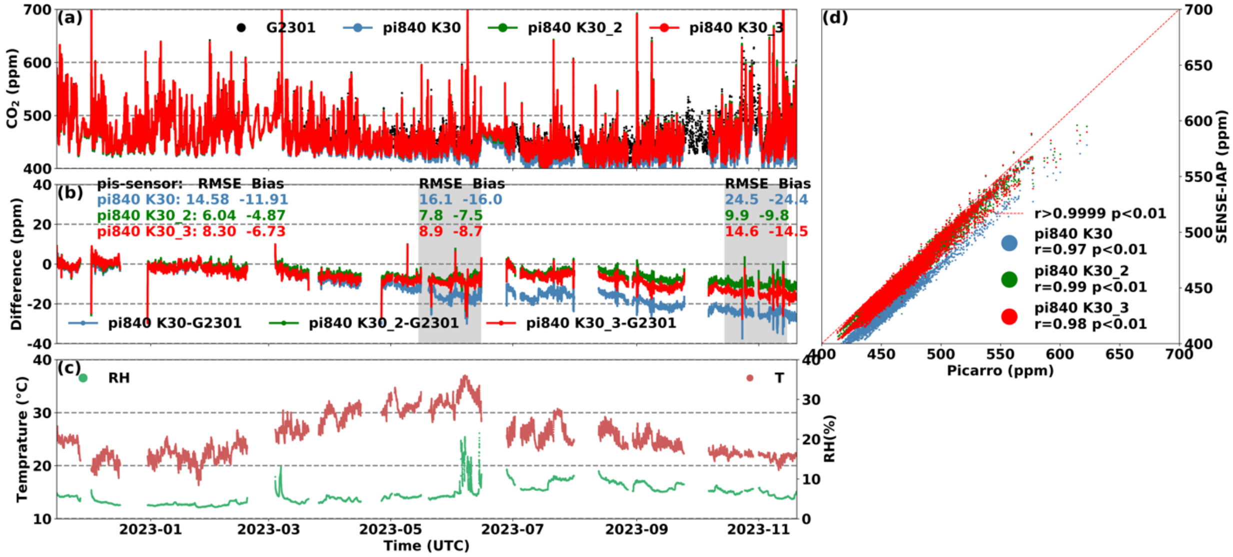

Figure S8: (a) Comparison between the hourly CO_2 concentrations measured by the three sensors on the SENSE-IAP and the Picarro system at Beijing-IAP from November 2022 to November 2023; (b) the time series of ΔCO_2 before long-term drift calibration; (c) the temperature and RH of the SENSE-IAP, (d) scatter plot of the SENSE-IAP and Picarro.

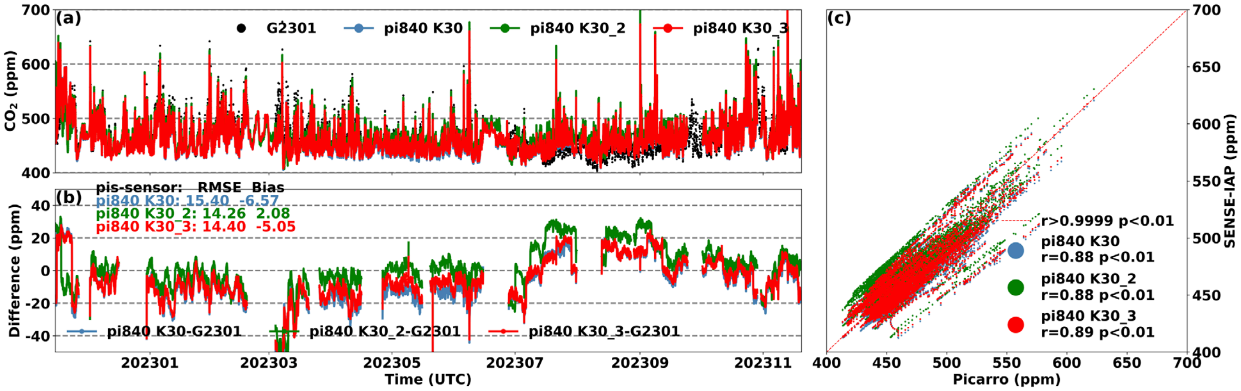

Figure S9: (a) Comparison between the hourly CO_2 concentrations measured by the three sensors on the SenseAir and the Picarro system at Beijing-IAP from November 2022 to November 2023; (b) the time series of ΔCO_2 before long-term drift calibration; (c) scatter plot of SenseAir and Picarro.
